# Delivery of genome editing tools by bacterial extracellular vesicles

**DOI:** 10.1111/1751-7915.13356

**Published:** 2018-12-13

**Authors:** Yue Liu, Eddy J. Smid, Tjakko Abee, Richard A. Notebaart

**Affiliations:** ^1^ Food Microbiology Wageningen University and Research PO Box 17 6700 AA Wageningen The Netherlands

## Abstract

Here we propose to use bacterial cells as factories that generate EVs harboring both the RNP complex (or any other CRISPR‐mediated tool) and a specific ligand molecule. The ligand would allow specific binding of EVs to cells with the matching receptors, adding a level of specificity to the delivery, hence stimulating precise genome editing.

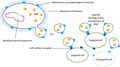

The production and release of extracellular vesicles (EVs) are a common property of cells spanning all domains of life (Deatherage and Cookson, 2012). These EVs enable the transport of various compounds, such as nucleic acids, proteins and viral particles, and are therefore potentially involved in cell communication, competition and survival (Kim *et al*., 2015; van Niel *et al*., 2018). From an application point of view, EVs could play an important role in health and disease, for instance in the development of novel strategies for vaccination and phage therapy (Liu *et al*., 2018). With respect to the latter, it has been shown for *B. subtilis* that phage‐resistant strains became susceptible for phages as a result of fusion with EVs carrying phage receptors (Tzipilevich *et al*., 2017).

Extracellular vesicles carry cargos both on or embedded in the membrane, as well as inside the lumen. Extracellular vesicles offer the enclosed cargos protection against (non)enzymatic degradation and are thus essential for exchange of RNA, proteins and other molecules that are prone to degradation (Mashburn‐Warren and Whiteley, 2006; Tsatsaronis *et al*., 2018). Moreover, EVs have the potential to facilitate the delivery of molecules that are generally excluded from entering target cells due to size, charge or hydrophobicity. Here, we argue that the production of EVs may also be harnessed to produce cell‐specific delivery systems for genome editing tools, such as the CRISPR toolbox including Cas9, Cas12a and base editors (Gaudelli *et al*., 2017; Knott and Doudna, 2018). One of the most important research directions in applying genome editing tools, especially in the context of medical applications to correct genetic disorders or to combat pathogens, is the delivery of the CRISPR toolbox to the right position in the human body or to the pathogens (Doudna and Charpentier, 2014; Sander and Joung, 2014). It is hereby preferred to deliver the nuclease enzyme (e.g. Cas9) in complex with the gRNA, the so‐called CRISPR‐Cas9 RNA–protein complex (or ribonucleoprotein complex; RNP), which after uptake allows precise genome editing. Currently, efforts are made to deliver RNP complexes via virus‐like particles, receptor‐mediated endocytosis or osmocytosis (D'Astolfo *et al*., 2015; Liu *et al*., 2015; Yin *et al*., 2016; Chaverra‐Rodriguez *et al*., 2018).

We propose to extend the delivery toolbox for genome editing approaches by bacterial EVs. The idea is to use bacterial cells as factories that generate EVs harbouring both the RNP complex (or any other CRISPR‐mediated tool) and a specific ligand molecule. The ligand molecules could be cell membrane‐anchored proteins. The presence of such ligand molecules on the surface of the EV would allow specific binding to cells with the matching receptors followed by fusion of EVs and cells and delivery of cargo. This adds a level of specificity to the delivery, hence stimulating precise genome editing. In practice, genes encoding a nuclease (e.g. Cas9) and gRNA will be introduced in the appropriate EV‐producing bacteria. These genes and gRNA should be constructed downstream of strong promoters to allow high production of the RNP complex. In the same bacteria, the DNA sequences of the ligand and a native bacterial membrane protein can be fused together to allow display of the ligand on the cell membrane. When EVs are generated from the cell membrane, they contain the ligand on the surface and most likely enclose the RNP complexes in the lumen, as the complexes are expressed in great abundance in the cytosol.

It should be noted that the above proposed model is most applicable for Gram‐positive producer bacteria, as they, in contrast to Gram‐negative bacteria that have an additional outer membrane, only have a cytoplasmic membrane acting as the origin of EV generation, and the membrane is in direct contact with cytosolic components to achieve engulfment of cargos (Toyofuku *et al*., 2018). Moreover, toxicity caused by lipopolysaccharides (LPS), which hampers the application of Gram‐negative bacteria (Acevedo *et al*., 2014), is also not of concern for EVs generated from Gram‐positive bacteria. In Gram‐negative bacteria, the outer membrane is the well‐known origin for EV generation, but such EVs generated cannot easily pack cytosolic components (Bonnington and Kuehn, 2014). Recently, evidence has been provided that inner‐membrane fragments can also be part of EVs, resulting in the release of merged inner–outer membrane vesicles (Pérez‐Cruz *et al*., 2015). Using this type of EVs for delivering genome editing complex is challenging, as the chance for all the required components to end up in the same EV could be considerably low.

In spite of the advantages that Gram‐positive bacteria can offer for the proposed application, it should be noted that Gram‐positive bacteria naturally produce EVs in lower quantity than the Gram‐negative. Nevertheless, knowledge on the biogenesis of EVs has been accumulated and pointed out ways to increase EV release. For example, explosive cell lysis is a source of EVs (Turnbull *et al*., 2016); cell wall degrading enzymes also play a role in EV release by weakening or penetrating the major barrier for EVs to escape (Toyofuku *et al*., 2017). By employing these enzymes, or inducing explosive cell lysis, EVs can be harvested in large amounts also from Gram‐positive bacteria.

When the proper bacterial cell factories are chosen, a crucial point to achieve the proposed cell‐specific delivery system is a justified design of the ligand molecule. This molecule should be chosen/designed in such a way that it binds specifically to the target cells without triggering unwanted signal cascades or immune responses. When the ligand molecule is fused to the bacterial membrane protein, proper folding of the ligand should be guaranteed for efficient targeting effect. With knowledge from multiple research fields, such as bioinformatics, biochemistry, microbiology and pharmacology, proper design of such a delivery system can be achieved.

We anticipate that the proposed strategy could pave the way for the production of cell‐specific delivery systems by exploiting bacteria as cell factories of both the genome editing tools and EVs. In our vision, this strategy can lead to the production of relatively low‐cost but high‐efficiency, high‐specificity delivery systems of genome editing tools for biotechnological and medical applications ranging from steering metabolite production to fighting pathogenic bacteria or correcting human genetic disorders.
